# A short simple tool to measure the impact of food allergy on patients in routine clinical practice; the Food Allergy Quality of Life Questionnaire, Parent Form 10 (FAQLQ-PF10)

**DOI:** 10.1186/2045-7022-5-S3-P7

**Published:** 2015-03-30

**Authors:** Donal Moynihan, Eimear Morrissey, Lianne Soller, Kasia Pyrz, Bertine Flokstra-de Blok, Anthony E J Dubois, Jonathan O’B Hourihane, Audrey Dunn Galvin

**Affiliations:** 1 University College Cork, Cork, Ireland; 2 McGill University, Montreal, Canada; 3 Groningen University, Groningen, The Netherlands

## Aim

Food Allergy is suboptimally managed, and the future risk for patients with regard to health status and quality of life is rarely evaluated in clinical contexts. Existing assessments do not meet physicians’ needs for a short simple tool to measure the impact of food allergy on patients health related quality of life (HRQL) in routine clinical practice.

We aimed to modify the well-validated Parent Form of Food Allergy Quality of Life Questionnaire (FAQLQ-PF) to develop a short, reliable, valid, one scale measure.

## Methods

In Phase 1, the FAQLQ-PF was completed by 61 parents in Cork University Hospital (CUH).

In Phase 2, the FAQLQ-PF and the FAQLQ-PF10 was completed by 48 parents in Cork University Hospital. The data was subjected to psychometric analysis, including an assessment of scale and item reliability and construct validity.

## Results

Children (62% male) were aged between 6 months and 13 years in both phases of the study. The new short version FAQLQ-PF10 has 10 questions that assess the impact of food allergy on health related quality of life. Analyses demonstrated high reliability (α = .89). Bartlett’s test of sphericity, *x*^2^(45) = 263.47, *p* ≤ .001, showed good correlation between items. The Kaiser-Meyer-Olkin(KMO) measure verified sampling adequacy(0.84). Two components had eigenvalues over the Kaiser’s criterion of 1 and explained 64% of the variance in the impact of food allergy on HRQL, demonstrating excellent construct validity.

## Conclusion

We have shown that the new FAQLQ-PF10 is a short easy to use precision instrument that is both reliable and valid. It may assist primary care physicians to capture the impact of food allergy on patients’ daily lives and wellbeing, improve long-term management and help to provide optimal care for patients with food allergy.

